# LabVis: usability testing of a prototype tool for integrating timeline graphs and clinical notes

**DOI:** 10.1186/s12911-025-03173-7

**Published:** 2025-09-26

**Authors:** Torbjørn Torsvik, Andreas Brun, Bjørn Ståle Benjaminsen, Aslak Steinsbekk

**Affiliations:** 1 Department of Neuromedicine and Movement Science, Faculty of Medicine and Health Sciences, Norwegian University of Science and Technology, Trondheim, N-7491 Norway; 2 Department of Public Health and Nursing, Faculty of Medicine and Health Sciences, Norwegian University of Science and Technology, Trondheim, N-7491 Norway

**Keywords:** Clinical data, Data visualization, Electronic health record, Laboratory medicine, Usability testing, Temporal reasoning

## Abstract

**Background:**

Effective patient care requires clinicians to develop a comprehensive understanding of a patient’s medical history, a task made challenging by the complexity of electronic health records (EHR). Timeline-based overviews have the potential to simplify this process, but proposed solutions often lack seamless integration between graphical summaries and free-text notes. The purpose of this study was to evaluate a prototype (LabVis) that uses temporal relationships to help clinicians quickly contextualize notable lab graph findings by enabling fast access to clinical notes from the same time period.

**Methods:**

LabVis was developed based on input from experienced clinicians and existing literature. It features interactive timelines and semantic zooming to manage data density, along with tools for connecting findings in graphs to free-text notes. The prototype was tested with 10 physicians. Each session included task-based interaction with LabVis, direct observation, a System Usability Scale (SUS) questionnaire, and a semi-structured interview. Observational data were systematically reviewed to identify and rate usability issues, and interview transcripts were analyzed using Systematic Text Condensation.

**Results:**

Participants reported high usability, with an average SUS score of 83. Timelines were said to help quickly identify key events, while integrated document navigation enabled efficient access to detailed notes. While participants emphasized the need for greater customization, there were no uniform suggestions for how to achieve this without compromising usability.

**Conclusions:**

LabVis demonstrates efficient strategies for linking timeline-based overviews with free-text notes. The study underscores the importance of intuitive navigation, prioritization of key information, and customizable views for clinical workflows. These insights can be leveraged to design EHR systems that better support clinical decision-making. Further research is needed to explore how best to ensure customizability without compromising usability in a real-life setting.

**Supplementary Information:**

The online version contains supplementary material available at 10.1186/s12911-025-03173-7.

## Background

To effectively treat patients, clinicians must develop a comprehensive overview of the patient’s history, drawing on a wide range of information often gathered from their medical records [1]. As patients age and more treatment options become available, their medical records can quickly become unmanageably large, making it time-consuming and labor-intensive to compile a coherent overview [2, 3]. Significant efforts have been made to research how various visualization techniques can better support clinicians in finding information [4]. Still, EHRs are often noted for limited alignment with clinical workflows. Problems such as cluttered layouts and inadequate strategies for handling large volumes of information can impede clinicians’ ability to access and interpret key data [5].

Laboratory tests, such as blood sample analyses, are essential for diagnosing and monitoring many diseases. In EHRs, these results are mostly presented as reports or tables [6]. However, due to their quantitative nature, test results can also be displayed in more visual ways. For example, EHRs have been known to include at least some charting functionality, often presenting time series of test results as line graphs [7]. Such graphs have been shown to give a concise overview of trends and changes in data, revealing patterns that may be less apparent in tables or static reports [8, 9].

However, graphs viewed in isolation often have limited utility. Laboratory test results typically acquire clinical relevance only when considered within a broader medical context [10]. Advanced visualizations can enhance the interpretation of lab results by presenting them on timelines alongside related data that may influence them [11, 12]. These visualizations can provide a clear, chronological view of events and show how different parameters fluctuate and affect each other over time.

While timeline-based designs hold promise, implementing them in real-world clinical practice is challenging. Patient records often contain large volumes of heterogeneous data that are unevenly distributed over time, making timelines prone to clutter and inefficient use of space [13–15]. Additionally, a substantial amount of important information in medical records cannot be easily presented in timeline-oriented displays. In routine clinical practice, clinicians often rely on free text documentation, which contains qualitative nuance and contextual richness that is difficult to represent visually [16, 17].

Despite the widespread use of free text for presenting and understanding patients’ medical histories, there is a significant lack of attention given to effectively integrating this data with timeline-based overview displays. In a systematic review, Ramalho et al. note that free text is often excluded in single-patient overview displays, hypothesizing that this exclusion is due to the lack of commonly used visualization techniques that mixes well with qualitative data [18]. In examples where free text was included, Ramalho notes that authors often used small, simple datasets, and it was unclear how the visualization would function with larger amounts of data. In a systematic review of the state-of-the-art in interactive EHR visualization, Wang et al. argue that the combination of both quantitative and qualitative information in EHRs presents significant scalability challenges for large patient records [19]. They emphasize the need to develop interaction techniques to effectively explore and navigate heterogeneous patient records, particularly in the temporal dimension.

Sultanum et al. [20, 21] argue that EHR interfaces should center on free-text clinical notes. While unstructured text poses scalability challenges, they caution against replacing it with visualizations. Without seamless access to the underlying notes, such tools may fail to gain user trust, resulting in limited adoption in real-world clinical practice. Instead, Sultanum advocates for designs in which free-text content remains central, with visual aids integrated to support and contextualize the text rather than functioning as standalone features. Similarly, a qualitative study found that physicians often use line graphs to detect anomalies in lab results but usually turn to free-text notes to understand the clinical context [10]. While effective for generating insights, this approach was described as time-consuming and therefore used only selectively.

Building on these insights, a computer program called LabVis was developed. LabVis is a prototype designed to better integrate line graphs of laboratory results with free-text clinical notes. LabVis implements simple yet scalable functionality to make it easier to locate notes by time. Grounded in the assumption that events occurring close in time are often thematically related, it enables users to move fluidly between visual trends and their clinical context. LabVis was created to test these concepts in practice and to inform future design.

The purpose of this study was to evaluate a prototype (LabVis) that uses temporal relationships to help clinicians quickly contextualize notable lab graph findings by enabling fast access to clinical notes from the same time period.

## Methods

### Description of LabVis

The LabVis user interface was programmed in Java 17, using the JavaFX 20 graphical framework.

**Fig. 1:**
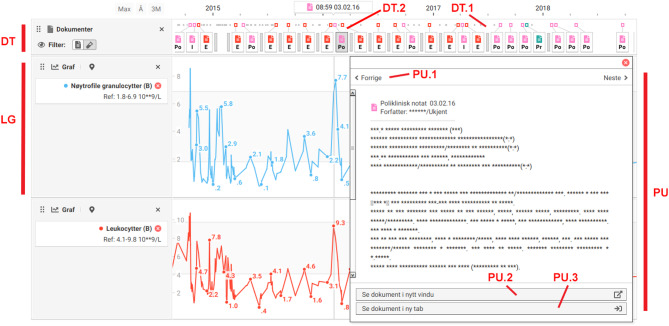
A view which includes a timeline panel with three timelines: two lab graphs (LG) and one document timeline (DT). All timelines share a common x-axis, displayed at the top.The lab graphs display time series of lab test results as simple line graphs. The y-axis represents test values, with reference ranges indicated by gray rectangles in the background. In the document timeline (DT), records are represented as glyphs. Each glyph is depicted as a rectangle containing an icon and letters indicating the document type. To optimize screen space, glyphs may be slightly horizontally displaced. Every glyph is connected to an anchor, represented by a small colored rectangle, indicating its actual position. Gray boxes (DT.1) represent one or more records that have been condensed due to space constraints. The lab graphs show a spike in values around February 2016. A glyph representing an outpatient clinic note (DT.2) from around that time has been clicked, triggering a popup (PU) that displays the full record in a paginator. The paginator allows browsing between documents using “prev/next” controls (PU.1). Users can use buttons at the bottom of the popup to either open the document in a new window (PU.2) or add a document panel showing the document to the view (PU.3)

**Fig. 2:**
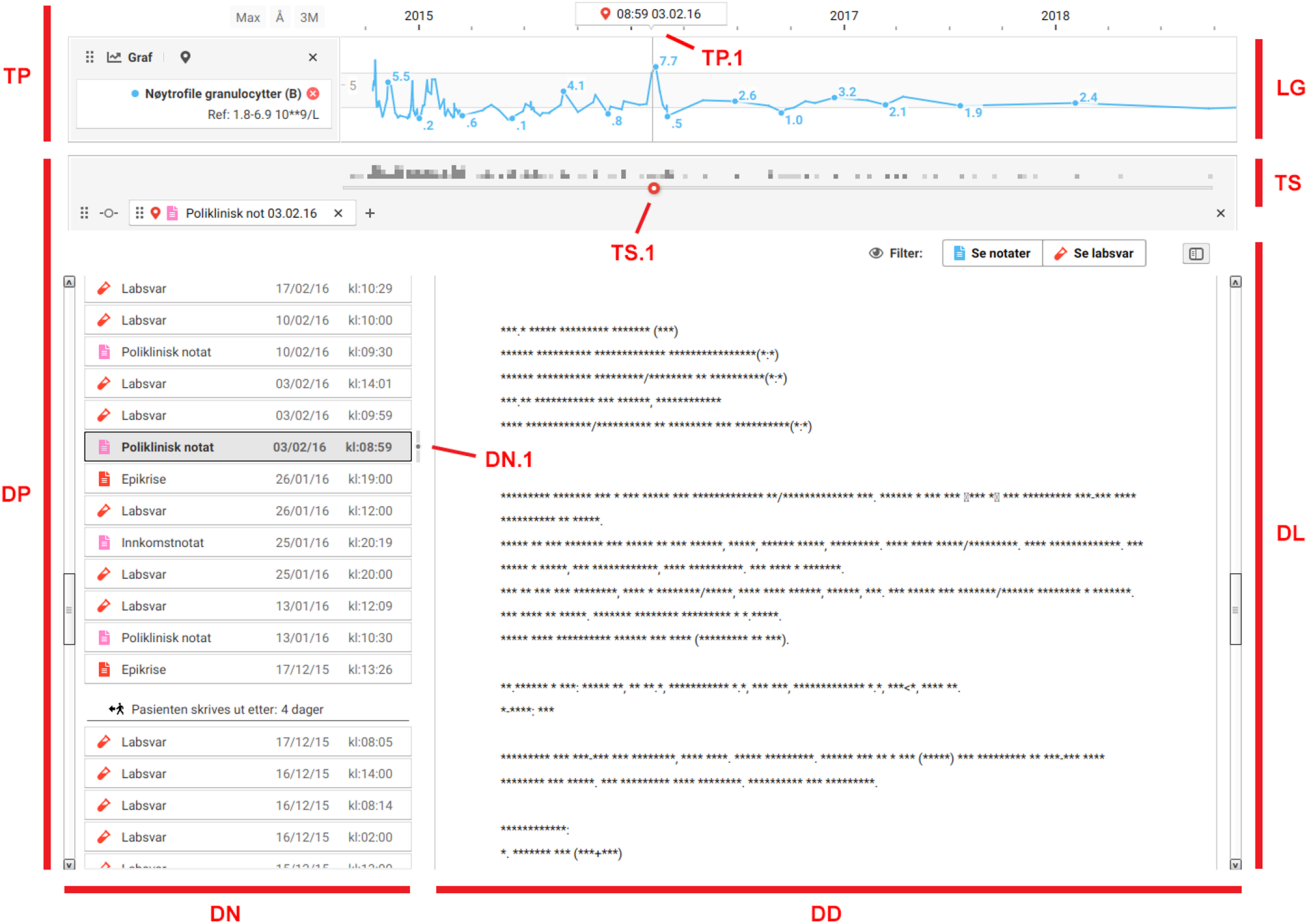
A view consisting of a timeline panel (TP) on top and a document panel (DP) below. The document panel consists of a document list (DL) and a time slider (TS). The document list consists of a document display (DD) showing full records, and a document navigator (DN). The document navigator displays available records as vertically stacked buttons, each labeled with a title and time of recording.The timeline panel contains one lab graph (LG). A spike in values can be seen around February 2016. By dragging the time slider handle (TS.1) to the area in question, the document list is adjusted to show documents from around the same time. The record currently displayed in the main window is visually highlighted in the document navigator (DN.1), represented as a marker on the timeline panel (TP.1), and aligned with the position of the time slider handle

The LabVis user interface features two types of main panels. A **timeline panel** (Figs. 1 and 2,TP) and a **document panel** (Fig. 2,DP). Panels can be shown either in isolation or together in a **view** (Figs. 1 and 2).

The **timeline panel** (Figs. 1 and 2,TP) is based on the LifeLines concept introduced by Plaisant [22]. The panel can hold one or more vertically stacked timelines. There are two types of available timelines, **lab graphs** (Figs. 1 and 2,LG) and **document timelines** (Fig. 1,DT). Timelines can be added or removed dynamically. Data on timelines is plotted linearly by time on a shared x-axis. The timeline is both zoomable and pannable.

Document timelines can present all records in the EHR. Similar to LifeLines, records are displayed over time as clickable glyphs. To reduce visual clutter when many records are shown, LabVis uses a variant of semantic zooming. When space is limited, excess records are condensed into grey boxes that indicate the presence of additional, filtered-out content (Fig. 1,DT.1). As users zoom in or out, elements are dynamically revealed or condensed as space allows. This approach allows access to details at any zoom level.

The document timelines use an internal ranking system to identify the most important records. Notes are ranked first by type, prioritizing categories like discharge summaries and procedural notes, and then by text length. Lab reports are ranked based on the number of abnormal test results, followed by the total number of tests in the report in descending order. When determining which records to display or condense, LabVis prioritizes those with the highest rankings.

The document panel contains a **document list** (Fig. 2,DL) and a **time slider** (Fig. 2,TS). The document list displays all documents and lab reports in a continuous, chronologically ordered list. The document list presents full records in the **document display** (Fig. 2,DD). A **document navigator** (Fig. 2,DN) provides an overview of available records. The time slider is located at the top of the document panel to facilitate coordination between the document panel and the timeline panel. The time slider features a density graph that plots all records in the document list along the same time coordinates as the timeline panel. A handle on the slider is positioned according to the same coordinates to indicate the time of the record currently shown in the document display. The time slider allows users to navigate the document list by dragging the slider handle (Fig. 2,TS.1). When dragging the handle, the list adjusts to display the record closest to the time corresponding to the handle’s new position. Similarly, when navigating the list, the handle in the slider dynamically repositions to reflect the newly selected record’s time of recording.

### Research design

**Fig. 3:**
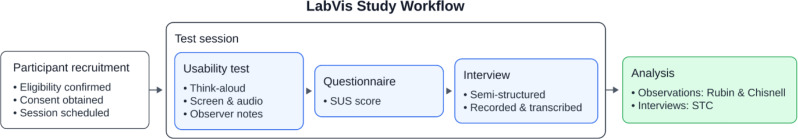
Study design

This study employed a primarily qualitative research design, centered on a usability test that included observations and semi-structured interviews, and was complemented by a System Usability Scale (SUS) questionnaire. An overview of the study design is presented in Fig. 3.

#### Setting

Data collection took place at the Laboratory Medicine Clinic at St. Olavs Hospital (SOH), Trondheim, Norway. Physicians at the clinic specialize in various disciplines within laboratory medicine and usually have experience in both primary and specialist healthcare. SOH is a university hospital with around 1,000 beds. SOH primarily uses an EHR to document and display patient health information, including laboratory test results.

#### Sample

Eligible participants were physicians either with a specialization or in specialist training, and employed at the Laboratory Medicine Clinic. The selection process aimed to ensure variation in gender and clinical experience. Potential candidates known to the authors were invited to participate via email, which included study details and a consent form. Sessions were scheduled for those who agreed. Consent forms were signed either in advance or at the start of each session. Each session lasted between 1 and 2 h. A total of 10 physicians participated. Participant characteristics are summarized in Table 1.

**Table 1:** Characteristics of participants

Gender	4 men, 6 women
Age	20–26 years: 1, 30–39 years: 5, 40–49 years: 4
Clinical experience	Mean 11 y, Min 1, Max 24

#### User test and data collection

LabVis was evaluated through a series of ten user tests conducted between January and May 2022, each followed by an individual interview. The tests aimed to assess usability and explore how clinicians interact with the system.

During user testing, participants used the prototype to interpret laboratory results from a real patient case. The patient was recruited by an independent physician unaffiliated with the research team, who identified a case involving a complex medical history where laboratory results played a pivotal role in assessing clinical progress. The patient provided informed consent for their data to be used in the evaluation. The case presented in the prototype included more than 450 clinical records and more than 2,000 individual lab results.

A protocol was developed to guide the testing process, including what to observe and which questions to ask. Participants were encouraged to think aloud, and both audio and screen activity were recorded. Each session was supervised by one researcher who provided assistance as needed, while another observed and documented relevant events using an observation guide. Particular attention was given to incidents where participants reported challenges. In such cases, participants were prompted to explain the issue, and possible causes and solutions were discussed when relevant. All observations were timestamped and documented, including descriptions of the problems, likely causes, and potential solutions where applicable.

In the first two sessions, participants were asked to configure views independently. Following initial feedback indicating that this approach was challenging within the time available, the protocol was revised to include a sequence of predefined views. In the revised sessions, participants were guided through a sequence of predefined views, progressing from simple to more complex. These views were designed to gradually introduce the functionality of LabVis. Participants still had the option to adjust the views but were no longer required to configure them from scratch.

In the initial, simpler views, participants completed tasks and answered questions designed to familiarize them with the basic features of LabVis (e.g., “Zoom in to this section of the chart,” “What is the highest calcium value shown in this chart?” and “When was the most recent test result recorded?”). As the user tests progressed, the views became more complex, and participants were asked to make broader clinical assessments. These included identifying possible medical explanations for anomalies in the graphs and forming an overall impression of the patient’s medical history (e.g., “Do you notice any results of particular interest in this chart?”, “What might explain this pattern?”, and “What is your overall impression of this patient?”). The complete user test guide is provided in Supplementary File 1.

### Questionnaire

Participants completed a SUS questionnaire [23] immediately after the user test. SUS is a widely used, standardized tool for evaluating system usability, consisting of ten statements rated on a five-point scale from “Strongly disagree” to “Strongly agree”. Responses are converted into a score from 0 to 100, with higher scores indicating better usability. SUS scores over 81 fall within the 90th percentile and correspond to an A grade, while scores below 68 are generally considered below average, indicating that the system may need improvement.

### Interview

After completing the questionnaire, a focused interview [24] was performed following a semi-structured interview guide. Participants were first asked about their general view of LabVis. Thereafter, three main topics were introduced if not talked about spontaneously: (1) the experience of having data connected by time, (2) the experience of having standardized views, and (3) the potential usefulness of LabVis in the participants’ daily work. Audio from interviews was recorded and transcribed verbatim. The full interview guide is available in Supplementary File 1.

#### Analysis

Observations were analyzed using the Rubin and Chisnell framework [25], which rates usability issues on a scale from 1 to 4. A score of 4 indicates a problem that renders a feature effectively unusable, while a score of 1 reflects a minor, cosmetic issue. Issues were first rated independently and then reviewed collaboratively. Similar problems were grouped, and representative examples were included to ensure empirical grounding.

Interviews were qualitatively analyzed using Systematic Text Condensation, as described by Malterud [26]. Systematic Text Condensation consists of four iteratively performed steps: **Step 1: Overviewing** – Authors reviewed the entire dataset and identified a set of themes. **Step 2: Coding** – After collaboratively defining key themes, inclusion criteria were developed. The dataset was then revisited, with text snippets being sorted into the appropriate themes. **Step 3: Condensing** – The text snippets were synthesized into constructed statements that represented the grouped themes. **Step 4: Synthesizing** – These statements were further refined into descriptions, illustrated with descriptive quotes, and checked against the original data to ensure accurate representation. Throughout the process, each step was revisited multiple times, refining themes, coding rules, and synthesized statements as understanding of the dataset developed.

## Results

### Usability

Analysis of the observed usability issues revealed that the majority were rated as moderate or minor, with no features deemed completely unusable. The most frequent problems involved navigation between views and configuring them by adding, removing, or adjusting panels. These issues were no longer reported after the test protocol was revised. Additional recurring themes included missing interface elements and requests for enhanced functionality. In particular, the absence of a free-text search feature and the need for more fine-grained filtering were frequently noted.

The System Usability Scale (SUS) results indicated high usability, with LabVis achieving an overall mean score of 83 across all ten tests (median 85, range 55 to 100), equivalent to an A grade [23]. Notably, the first two user tests yielded significantly lower scores. After adjustments to the user test protocol, the final eight tests showed marked improvement, with an average score of 90, corresponding to an A + grade.

### Interview analysis

#### Timelines provide an overview

During testing, participants were observed using timelines to rapidly assess the patient’s clinical status. The density graph in the time slider (Fig. 2,TS) highlighted periods with a high number of records. Some noted that this gave them an overall impression of the patient’s medical history since frequent healthcare contact often indicated high disease activity. Participants also found it helpful to observe the duration of follow-up and the evolution of contact frequency over time. The lab graphs offered comparable insights, highlighting periods of frequent testing or significant fluctuations in test results.

If you have a chronic patient, you can see that here it was very dark [pointing to a dark area on the density graph], so a lot has happened. Then you can meet the patient and say that things are better now, but two years ago they were not. You can know what to focus on when meeting the patient.

It was commented that timelines were of limited utility when viewed in isolation. Several participants noted that the density graph, in particular, lacked sufficient granularity. Participants emphasized that they needed access to individual events to make meaningful assessments.

I don’t know how useful it can be. It should rather make it easier to find the exact information you’re looking for.

The system could display several timelines simultaneously. Some participants found this useful. For example, regarding lab results, different tests sometimes had to be interpreted together. Participants reported that when multiple trendlines were plotted on the same timeline, this facilitated the identification of covariation.

There can be other test parameters that follow each other, so it’s important to see them in context. For example, with thyroid diseases, when we look at how one hormone controls the value of another.

Participants explicitly stated that combining the lab graph with the document timeline was beneficial. Some explained that a common strategy for interpreting lab graphs was to first analyze them alone. If they found abnormal lab results, they would go to the document list and search for documents from the same period, which could often provide an explanation. Having a document timeline linked to the lab graph was reported to make it easier to locate such documents.

It’s very convenient to have notes related to the timeline. It’s important to see what has happened, and when things were done. To get an overview of the sequence of events, it’s very neat. It helps to get an overview in the shortest possible time.

#### Finding context

Participants could explore full records in the document timeline by clicking on glyphs (Fig. 1,DT.2), which triggered a popup. Although generally appreciated, several participants reported that the popups lacked essential contextual information available in the document list. When an interesting document was found, participants often found it helpful to read other documents from the same time period. Since the document timeline filtered out many records, it was harder to get a full overview of single events. Viewing documents in the list provided a clearer sense of how events unfolded.

Yes, it’s interesting to see journal notes in relation to lab values. But in a real medical record system, it would probably be desirable to just flip through journal notes too.

Participants could zoom in on a specific timeframe within the document timeline to view all documents from that period. However, this often made them feel like they were losing track of the overall context.

When you zoom in, it’s fine, you have the number of days, but then there’s a lot of side-scrolling. In that sense, it seems safer to go through a list showing which text sections you can click on; then you don’t miss anything.

Many participants expressed a preference for views that integrated both a timeline panel and a document panel (Fig. 2). When these were displayed simultaneously, participants could navigate the document list to areas of interest on the timeline by dragging the time slider handle (Fig. 2,TS.1). Some commented that this was easier than using the popup.

I don’t have to think about the documents; they just appear [when navigating using the time slider]. I don’t have to click on them. Fewer clicks.

Participants noted that the time slider also added value by providing navigational cues during normal browsing of the list. For patients with extensive records, excessive scrolling to locate older entries often made it difficult for participants to keep track of their position. By establishing a clear connection between the timeline panel and the document list, the slider helped participants maintain awareness of which part of the overall history they were investigating.

Another thing about the slider is that you have the feeling that you have the whole medical record, which is different from a list. That’s a big difference.

#### Customization of views

Participants suggested that the views could be better tailored to their specific needs. For example, the document timeline was set to automatically filter out presumed unimportant documents when there wasn’t enough space to display them all. Some expressed a desire for more granular prioritization and filtering, so they could have greater control over which information was displayed.

Maybe important events could have been marked somehow, like if something urgent happened. Something important for further treatment.

Many participants suggested predefined compositions of timelines and document lists tailored to specific clinical issues they frequently encountered. There was significant variation in the participants’ suggestions.

For example, if I were a geriatrician, I could imagine combining blood pressure with medications, or dementia assessments with medications. Based on what you’re interested in, there will be different things you look for.

At the same time, some participants pointed out that having too many available custom views could be problematic, as it might lead to uncertainty about whether others involved with the patient had seen the same information. They noted that it was advantageous for everyone collaborating on a patient to be presented with the information in the record consistently. Some also believed that it would be easier to absorb information when using familiar views.

It can be both a good and a bad thing. If you do things the same way, it’s easier to collaborate because you’re seeing the same thing.

I’m kind of against having too many options. It has to do with recognition; you need a familiar picture that you’re used to seeing.

Several participants suggested offering a set of general views as a starting point, which participants could customize by adding or removing information as needed. It was emphasized that broad views should be provided initially, allowing participants to narrow their focus, rather than starting with detailed views and expanding them. It was generally pointed out that such a system would have to be user-friendly and easy to configure.

I think there should be a standard starting point for everyone, and then you can add or remove things yourself. At the same time, the default view should show all information, instead of restricting the data beforehand.

## Discussion

Participants in this study reported that LabVis enabled them to easily navigate from overview timelines to underlying documents. Participants preferred to look at documents in the document list, because it allowed them to easily navigate chronologically between records. Although the participants appreciated using preformed views, they had conflicting opinions on how such views should be provided in a real-life clinical setting.

Consistent with other studies, participants valued the lab graph’s ability to provide quick overviews of large sets of lab results [8, 9]. Also in line with previous research, they noted that such overviews alone were typically insufficient to be truly informative [9]. To help participants connect graphical findings to relevant clinical details, LabVis incorporated a document timeline capable of presenting all available records in the patient’s chart, drawing on the LifeLines concept introduced by Plaisant et al. [22].

LabVis was evaluated using a realistic patient record containing hundreds of entries. While LifeLines-inspired timelines are widely studied, they rarely include documents and even less frequently attempt to visualize realistic data volumes [18, 27]. Notable exceptions include Doccurate [28] and HARVEST [29], which were designed to provide overviews of and access to large sets of clinical notes. Similar to LifeLines, as the amount of information increases, both systems rely on abstraction techniques to reduce timeline overcrowding. While this preserves readability, it obscures details and limits direct access to underlying data. Neither system supports a direct path from timeline to raw records, instead requiring navigation through intermediate layers. LabVis addresses this limitation by preserving a direct link from timeline to source documents, even when abstraction is applied, enabling single-click access to details at any zoom level.

When zoomed out to display hundreds of notes, only key records remained fully visible and interactable, while less relevant ones were abstracted into gray boxes. Although this approach resulted in an unbalanced view of the overall dataset, participants rated it highly, suggesting that they primarily needed the document timeline for locating and navigating to notes. This aligns with Sultanum’s recommendation of prioritizing the integration of free text notes when designing overview displays [20]. The extensive filtering may also have resulted in a simplified narrative, making it easier for participants to process. In contrast, testing with prototypes such as Doccurate revealed that some users feel less confident in their patient assessments when presented with a comprehensive overview of the medical history [28]. This suggests that having more background information can reduce confidence by introducing greater complexity. Conversely, limited information may foster false confidence by oversimplifying the situation. We did not evaluate the quality of the participants’ assessments, so this remains uncertain.

LabVis determined which notes to highlight in the document timeline using a fixed ranking system that prioritized certain note types, as described in Methods. This approach was informed by Hripcsak et al., who reported that some note types, such as discharge summaries and procedural notes, tend to be more impactful than others [30]. Participants considered this ranking reasonable but noted its limitations, expressing a need for more granular approaches that account for context-specific relevance. However, the lack of precision in the document timeline was not reported as its primary limitation. While participants appreciated the document timeline, they generally preferred using the document list in combination with the time slider. It was noted that when opening a particular note via popup, it was often useful to also see the notes written before or after. Although the popup allowed participants to browse sequentially through records, participants felt the document list provided a better localized overview. The stressed importance of such localized overviews aligns with the findings of Varpio et al., who emphasize that the chronological organization and interconnectedness of patient data are critical for constructing a coherent narrative and supporting clinical decision-making [31]. Clinical notes are usually written as the patient’s story unfolds, and must therefore often be viewed in their temporal context to understand how they fit into the broader narrative. When using the document list without the time slider, participants reported losing their sense of orientation when scrolling far down. Thus, coupling the document list with timeline panel proved useful not only for contextualizing graphs but also for navigating the document list, indicating a synergistic relationship between the two. The findings in this study suggest that the ability to navigate and remain oriented in time was considered more valuable than precise selection of individual notes.

During the initial user testing, participants were asked to configure their own views with minimal guidance. This proved to be overwhelming. The first participants spent considerable time trying to understand how to use the prototype and reported low usability. In response, the testing protocol was revised to include a sequence of predefined views. This change significantly improved task completion times and enhanced perceived usability.

Most participants were introduced to LabVis through a series of predefined views. While many expressed a desire to further customize the interface to suit their individual needs, opinions varied on how this should be achieved. Jensen et al. noted that overview displays work best for clinical tasks where information needs are consistent [32]. However, for complex patients, it is difficult to predict what information clinicians will need, making it difficult to design effective predefined displays. Participants echoed this concern, questioning whether pre-designed, problem-oriented views could balance broad applicability with manageable complexity. At the same time, those who used LabVis without preconfigured views reported poor usability when forced to configure them on their own. This highlights a central challenge: how to best integrate LabVis-like functionality into real-world systems. Cohen et al. similarly observed that while users benefit from control and transparency, too much freedom can reduce usability [33]. Striking a balance between automation and guided interaction remains key. Recent prototypes have explored using machine learning to generate tailored, problem-oriented views dynamically, showing improved performance and positive user feedback [34, 35]. Such approaches suggest potential pathways for translating LabVis concepts into practical applications.

### Limitations

Although participants reported high usability, it remains unclear whether they actually gained better clinical insights using LabVis as this was not measured. Additionally, participants were primarily exposed to the interface through pre-made views that highlighted LabVis’s functionality. The participants were from a single clinic at one hospital, which limits the generalizability of the findings. Other healthcare providers may engage with laboratory results differently. All participants were exposed to the same patient records, and those working with predefined views followed the same task sequence. While some informal sharing between colleagues was possible, it would likely have had little effect, since the tasks were exploratory and focused on interaction and usability rather than recall or performance.

## Conclusion

Using temporal relationships, LabVis enabled clinicians to navigate between lab graphs and clinical notes, making it easier to connect findings in lab graphs with their clinical context. This was enabled by graphs that provided quick overviews, directly linked to clinical notes from the same period, which supported rapid contextualization of abnormal or notable results. The document timeline was effective for overview, quick navigation and prioritizing records, while the more detailed document list offered a localized chronological overview that clarified the context of individual documents. Although preferences varied, some prefer customizable views, which highlights the challenge of balancing standardization with adaptability in complex clinical settings. Further research is needed to evaluate the utility of temporal linking of lab data and clinical notes in real-world EHR systems.

## Supplementary Information

Below is the link to the electronic supplementary material.

Supplementary Material 1

## Data Availability

Software, patient records, audio files, and transcripts from user tests and interviews cannot be shared, in accordance with the project protocol approved by NSD. The guide used for conducting user tests and interviews has been translated into English and is provided as supplementary material.

## Abbreviations

EHR: Electronic Health Record
SUS: System Usability Scale
SOH: St. Olavs Hospital
NSD: Norwegian Centre for Research Data
Sikt: Norwegian Agency for Shared Services in Education and Research
