# Supplementary material for: LabVis: usability testing of a prototype tool for integrating timeline graphs and clinical notes
Source: BMC Med Inform Decis Mak. 2025 Sep 26;25:337. doi: 10.1186/s12911-025-03173-7 (PMC12465496; doi:10.1186/s12911-025-03173-7)
Supplement: Supplementary file 1 — Supplementary Material 1 [file 12911_2025_3173_MOESM1_ESM.docx]

Interview Guide / User Test Guide

# User Test guide

Brief the interviewee about the project.

1. Personal Information:
   - Age
   - Workplace
   - How long have you been a physician?
   - How long have you worked here?

The participant is asked to complete a series of tasks in the prototype. By completing the tasks, the user receives a structured introduction to the prototype’s functionality. The participant goes through preconfigured views that provide insights into the tool's features. There are a total of seven preconfigured views as shown in the image under “Preconfigurations (Ferdigvalg)”.


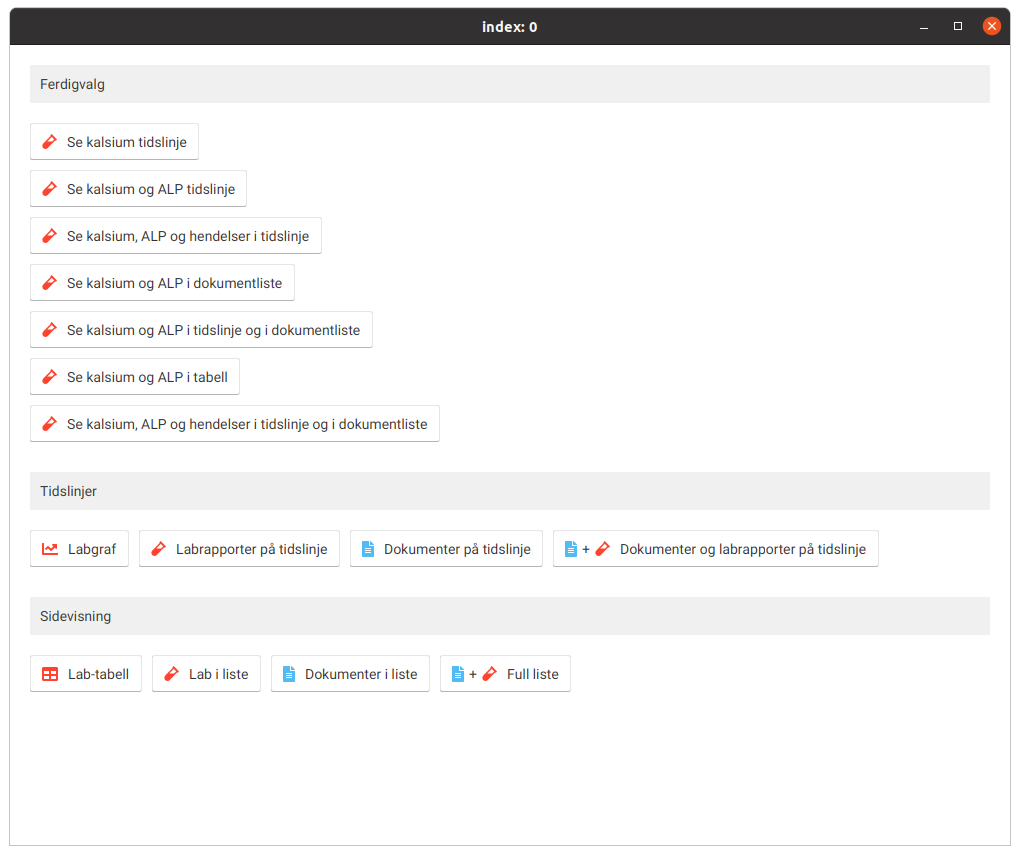


**Preconfiguration 1: View calcium timeline**

1. Examine calcium in the timeline:
   1. When was calcium highest/lowest?
   2. What are the highest and lowest calcium values?
   3. When is the first/last test result dated?

**Preconfiguration: View calcium and ALP in timeline**

1. Examine calcium and ALP in the timeline:
   1. What are your thoughts on what you see?

**Preconfiguration 3: View calcium, ALP, and records in timeline**

1. Examine events in the timeline:
   1. Zoom in on an event in the timeline and examine documents related to the event
      1. Explore the timeline filter for documents
      2. What do you now think about the patient’s history?

**Preconfiguration 4: View calcium, ALP, and document list**

1. Examine the document list:
   1. Search the document list for lab results on calcium and ALP
      1. Examine a lab result
         1. What do the different parameters mean?
   2. Filter notes to show only notes
   3. Filter notes to show only lab results
      1. Examine the note boxes between test results

**Preconfiguration 5: View calcium and ALP in timeline and document list**

1. Examine the relationship between the document list and the timeline:
2. The slider between the document list and the timeline moves as you navigate the document list — try it
   1. Possibly something about data density?
3. Use the slider to move through the document list — it can be dragged back and forth

**Preconfiguration 6: View calcium and ALP in table**

1. Examine the patient’s calcium and ALP — what do you think of this view?
   1. Try the link for a cell
   2. Try the link for a column
   3. Try the link for a row

**Preconfiguration 7: View calcium, ALP, and records in both timeline and document list**

Explain the overall picture and the possibility to add or remove elements
The participant is asked to freely try the program and reason through the patient’s history.

Ask the participant to look at analytes and test results, e.g., ALAT, creatinine, and platelets.

Ask the participant to explain changes and trends in the values.

*Panel of Potential Questions for Analytes:*

- Describe the lab results while thinking aloud. What do you see?
- What could be the cause of this?
- How would you proceed to investigate this further?
- Why are you doing this?

### **Interview guide:**

1. What did you like best?
   1. Why? Examples?
2. What did you like least?
   1. Why? Examples?
   2. Do you have any suggestions for improving the tool?
3. The program allows multiple ways to switch between views. What do you think of this?
   1. The program often provides the ability to see elements over time:
      1. Overview?
      2. Details?
   2. Can you imagine other strategies for navigating the patient record?
4. In the program, it’s possible to create custom views. What do you think of this?
   1. Can views be reused frequently?
   2. Used by many/few?
      1. Personal?
      2. By patient?
      3. Department level?
      4. Profession?
5. Do you think this tool could be useful in your work?
   1. Can you give examples of when it could be useful/not useful?
      1. If the participant doesn’t address this on their own:
         1. Do you think this system would help you make better decisions?
         2. Do you think this system would lead to better patient care?
         3. Do you think this system would reduce the chance of missing important information?
